# Aging and Psychological Well-Being: The Possible Role of Inhibition Skills

**DOI:** 10.3390/healthcare10081477

**Published:** 2022-08-05

**Authors:** Giulia Fusi, Massimiliano Palmiero, Sara Lavolpe, Laura Colautti, Maura Crepaldi, Alessandro Antonietti, Alberto Di Domenico, Barbara Colombo, Adolfo Di Crosta, Pasquale La Malva, Luca Rozzini, Maria Luisa Rusconi

**Affiliations:** 1 Department of Clinical and Experimental Sciences, University of Brescia, 25123 Brescia, Italy; 2 Department of Human and Social Sciences, University of Bergamo, 24129 Bergamo, Italy; 3 Department of Biotechnological and Applied Clinical Sciences, University of L’Aquila, 67100 L’Aquila, Italy; 4 Department of Neurology, Humanitas Gavazzeni Hospital, 24129 Bergamo, Italy; 5 Department of Psychology, Catholic University of the Sacred Heart, 20123 Milan, Italy; 6 Department of Psychological Sciences, University Gabriele D’Annunzio of Chieti-Pescara, 66100 Chieti, Italy; 7 Neuroscience Lab, Champlain College, Burlington, VT 05401, USA

**Keywords:** aging, psychological well-being, executive functions, inhibition, personal growth, mental health

## Abstract

(1) Background: Psychological well-being (PWB) is a multidimensional construct which is a key protective factor against chronic diseases in older adults. Numerous psychological and cognitive factors can influence older people’s PWB. However, while most studies have explored the effect of general cognition, only a few investigated the role of specific cognitive functions such as the efficiency of executive functions. Thus, the present study aimed to directly explore the relationships between PWB, age, and the core executive functions (namely working memory, flexibility, and inhibition). (2) Methods: A sample of 51 healthy older adults (age range: 60–88) were enrolled and tested both for PWB (by Ryff’s psychological well-being scale) and the core executive functions (by the Digit span backward, the Trail Making Test, and the Stroop test). (3) Results: Age and inhibitory skills are negatively correlated to the personal growth subscale of the PWB scale. Moreover, a hierarchical regression analysis showed that inhibition, but not working memory and flexibility, predicted the sense of continued growth and development (personal growth) of older adults, even after controlling for age and educational levels. Limitations and implications for future research and prevention interventions are discussed.

## 1. Introduction

Health has been historically defined as a state of complete physical, mental, and social well-being, not merely the absence of discomfort or disease. However, indices of health and well-being in our society have continued to focus mainly on distress, illness, and negative functioning for a long time [1]. Between 1989 [2] and 1995 [3], Carol Ryff merged different frameworks into a multilevel model of psychological well-being (PWB), where well-being was presented as a multidimensional and dynamic process that encompassed various psychological aspects. This model included six distinct dimensions of positive psychological functioning. The first dimension was described as the positive evaluations of oneself and one’s past life (Self-Acceptance); the second as a sense of continued growth and development as a person (Personal Growth); the third as the ability to have goals and a sense of direction for life and feeling that one’s life is purposeful and meaningful (Purpose in Life); the fourth as the ability to build relevant relationships with others based on trust and warmth, to experience strong feelings of empathy and affection and to create intimate relationships (Positive Relations with Others); the fifth as the capacity to effectively manage one’s life and the surrounding world (Environmental Mastery); and the sixth as the tendency to be self-confident and independent and to have a sense of self-determination (Autonomy).

PWB quickly became a central and widely investigated construct within health studies in different contexts, including the aging one. Research revealed that it plays a key role in the health of older adults by both exerting a significant risk reduction in chronic diseases and promoting longevity [4,5,6,7,8,9,10,11]. However, data regarding the developmental trajectories of the various subcomponents of PWB across the lifespan, especially during the later stages of life, do not always converge. For example, Ryff and Keyes [3] evidenced a decline with age in the Purpose in Life and Personal Growth subscales, whereas incremental scores were highlighted for Environmental Mastery and Autonomy. By contrast, a series of longitudinal studies showed that PWB dimensions follow a U-shaped trajectory [12]. Other studies, instead, showed that PWB does not consistently exhibit distinct age profiles or decline, with exceptionally small longitudinal age variations and far greater variations within different age groups [13,14]. Therefore, the relationships between age and different PWB dimensions appear to be complex and far from being completely understood.

Consistent with this complexity, several variables can also affect the relationship between PWB and age [15], such as biological [10], psychological (e.g., personality trait, [16]), and cognitive [17,18] ones. More in detail, focusing on cognitive functions, different studies (e.g., [19,20]) found that measures of global cognitive status were positively associated with PWB in older people samples. However, most studies have focused only on the relationships with general cognition [18,19] or fluid intelligence [19,20], without considering the possible role of specific cognitive abilities. In this vein, some models already suggested the involvement of higher cognitive functions in perceived PWB [21], while other recent studies suggested the specific role of executive functions (EFs) [22,23,24].

EFs refer to a group of adaptive, top-down cognitive control processes involved when subjects are engaged in a task and automatic responses are not allowed or possible [25]. There is a general agreement about three core executive functions (e.g., [25,26,27]): working memory, cognitive flexibility, and inhibition, which includes inhibitory control processes, including self-control and interference [25]. From these core functions, higher-order EFs are built, including planning and fluid intelligence, which encompasses reasoning and problem solving [28]. EFs are generally considered as skills essential for both mental and physical health [25,27]. 

Some specific models stressed that PWB might depend not only on past experiences and interpersonal comparison, but also on the intervention of complex cognitive abilities for the balance between subjects’ personal aspirations and payoffs (i.e., “psychological accounting theory”) (e.g., [21]). These complex evaluations would certainly require the intervention of higher cognitive functions, such as EFs. Accordingly, previous studies showed that cognitive control strategies—depending on EF efficiency—support adaptive psychological processes such as self-reflection, emotion regulation, and convergence of cognitive, motivational, and emotional goals [29,30,31]. Moreover, Shamsabadi et al. [32] reported that working memory, cognitive flexibility, quantitative reasoning, and planning correlated to all measures of PWB, with the exception that quantitative reasoning and planning were not correlated to Purpose in life and Positive relations, respectively. Additionally, some research showed that the decline in executive control processes, such as perceptual speed or working memory, was associated with a decline in PWB as measured by the Ryff’s scale, particularly with aspects of well-being related to purpose in life, self-acceptance, and autonomy [33,34], and especially with eudaimonic aspects of PWB [33] in older people. This is not surprising given that cognitive control plays a key role in emotion regulation [35], even though it is also strongly affected by age [36]. 

In this vein, age-related changes in EFs—such as those occurring in working memory, inhibition, and shifting [37]—can underlie the decline in those dimensions of PWB that are more cognitively demanding, such as maintaining a sense of purpose in life in older adults [33]. Based on these premises, the present study aimed to explore the extent to which the core EFs (i.e., working memory, shifting, and inhibition) affect PWB, after controlling for age and education. The hypotheses were formulated as follows: (1) As age increases, a decline occurs in at least some PWB subscales (such as personal growth and purpose of life) [3] and in the core EFs [25,27]; (2) core executive efficiency affects older people’s perceived PWB after controlling for age and education (the less efficient EFs are, the lower PWB scores are).

## 2. Materials and Methods

### 2.1. Participants and Procedure

A total of 64 Italian healthy older adults were recruited before the COVID-19 pandemic. Data collection took place between November 2019 and February 2020. All participants underwent an assessment to be screened for the inclusion criteria: age ≥ 60 years-old; Montreal Cognitive Assessment (MoCA) ≥ 15.5 [38]; no history of vascular, neurological, or psychiatric disease. All tests were administered individually, in a quiet setting and in a single session that lasted approximately one hour.

Considering the inclusion criteria and the drop out individuals, that is, participants who did not complete the entire assessment battery, 51 older people were included in the final sample (mean age: 72.3 ± 7.23 yrs., age range: 60–88 yrs.; mean educational level: 10.1 yrs. ± 4.49, range: 2–21 yrs.; 31 women, 20 men). The study was conducted following the Declaration of Helsinki and informed consent was signed by all the participants. The research protocol and procedure were approved by the institutional ethical committee of the Catholic University of Sacred Heart in Milan (Commissione Etica per la Ricerca in Psicologia-CERPS, Approval Code: 10–21). 

### 2.2. Materials

#### 2.2.1. General Cognition

MoCA [38] is a cognitive screening test designed to evaluate general cognition. It is composed of 12 sub-tasks that evaluate different cognitive domains: attention, EFs, memory, language, abstraction, calculation, orientation, and visuo-constructional abilities (max score = 30). This test is widely considered a sensitive tool for detecting mild cognitive impairment [39].

#### 2.2.2. Core Executive Functions

The core EFs—working memory, shifting, and inhibition, as defined in the Diamond’s model [25]—were evaluated by means of the Digit span backward [40], the Trail Making Test (TMT) [41], and the Stroop test [42], respectively. 

In the Digit span backward participants were asked to repeat inversely a sequence of numbers spoken by the examiner at a rate of 1 per second. The sequences of numbers varied from 2 to 8 digits. The span is the last sequence of numbers correctly repeated at least once.

The TMT consisted of 2 parts: In part A, participants were asked to join, with a continuous line made with a pen, numbers from 1 to 25 arranged randomly on a paper sheet; In part B participants were asked to join alternately numbers and letters (e.g., 1-A, 2-B, etc.) without lifting the pen from the page. The score in both parts is defined by the time taken to finish the test correctly. Then, the difference between the score of part B and the score of part A is taken as an index of cognitive flexibility: the higher the score is (and thus the time spent on part B compared to part A), the lower the subject’s switching abilities are. 

In the Stroop test participants were asked first to read words (color names) displayed in black ink (part 1), then to name colored (blue, green, red) dots (part 2), and finally, to declare the name of the ink color of the words (written in blue, green and red) that can or cannot match the name of written words (part 3). This procedure allowed the examiner to compute three response times (part 1, 2, and 3). Then, the score of “time interference” (TI) was computed using the following formula: TI = T3 − (T1 + T2)/2 (see [43] for further information). In clinical neuropsychology, this is usually considered as an index of inhibitory abilities: the higher the interference time is, the lower the ability to inhibit automatic responses is. 

#### 2.2.3. Psychological Well-Being

PWB [43] is an 84-item self-report questionnaire with responses provided on a 6-point Likert-type scale (1 = strongly disagree to 6 = strongly agree). The questionnaire evaluates six subscales: Autonomy (AUT; internal consistency = 0.86; test-retest reliability = 0.21), Environmental Mastery (EM; internal consistency = 0.90; test-retest reliability = 0.31), Personal Growth (PG; internal consistency = 0.87; test-retest reliability = 0.78), Positive Relations with Others (PRO; internal consistency = 0.91; test-retest reliability = 0.81), Purpose in Life (PIL; internal consistency = 0.90; test-retest reliability = 0.81), and Self-Acceptance (SA; internal consistency = 0.93; test-retest reliability = 0.82) [44]. Subscale scores were computed by summing the 14 items. Higher scores indicate greater PWB.

## 3. Analysis and Results

Descriptive statistics of the assessed measures (mean scores and standard deviations) are reported in Table 1. 

We checked for normal distribution of data using the criteria suggested by West et al. [44] (± 2 of asymmetry and ± 7 of kurtosis). Almost all variables were normally distributed according to West et al.’s 1995 criteria. Only Autonomy scores were square root transformed to meet these normality criteria. Then, we ran parametric correlations and regressions analyses.

We exploratively performed t-tests to control for differences related to gender (see Table 2) and correlations to investigate the relationships between age, education, PWB and EFs (see Table 3).

Only one significant difference (t(49) = −2.15; *p* = 0.037) emerged between women (M = 64.9, SD = 8.12) and men (M = 59.55, SD = 9.75) in the Self-acceptance PWB scale.

The analyses showed that only one moderate negative relationship emerged between age and the Personal Growth subscale of the PWB scale. Moreover, the latter correlated negatively and moderately to the Stroop_TI (time interference) score. 

Finally, socio-demographic variables showed, in line with neuropsychological literature [41,42], correlations with some of the cognitive variables. More specifically, age showed a positive correlation with Stroop_TI and TMT_B-A (the higher the age of the subjects, the higher the time to accomplish both the tasks), while education was negatively correlated to both tasks (the higher the education is, the lower the time to accomplish both the task was).

Considering the results of the t-tests and correlation analyses, a hierarchical regression with the Personal Growth subscale of PWB as the dependent variable, age and educational level as the first control block (to control for the effect of these two socio-demographic variables) and the three core EFs as the second block of independent variables was run (see Table 4). 

The analysis showed that the first model with the independent control variables (age and educational level) was significant (F (2,48) = 3.38, *p* < 0.042) and the 8.7% of the variance. None of the control variables showed a statistically significant impact on the DV. After introducing the EF scores (i.e., Digit_back, TMT_B-A, Stroop_TI) as a second block, the model was still significant F (5,45) = 3.56, *p* < 0.05) and explained 20.4% of variance, that is, an additional 16% (R^2^ change = 0.160; F (3,45) = 3.34; *p* < 0.05). Only Stroop_TI resulted in being a significant predictor of the dependent variable (b = −0.487, *p* = 0.003).

## 4. Discussion

The present study aimed to investigate the influence of executive efficiency on perceived PWB in a sample of Italian healthy older adults. We hypothesized that age might negatively impact at least some components of PWB and, most importantly, that the core EFs efficiency (working memory, cognitive flexibility, and inhibition) might positively impact the perceived PWB of older people after controlling for age and educational level.

Analyses confirmed, at least in part, our hypotheses. On the one hand, our results replicated Ryff’s findings [3]: the PWB subscale Personal Growth was indeed the only dimension to show a negative correlation with age. As age increases, even within an older-adults-only sample, the perception of significant personal growth decreases. Ryff and colleagues [3] argued that this might happen because opportunities for continued growth, meaningful experiences, and development are limited for older persons. According to this claim, more recent evidence proved that older adults who engaged in more diverse activities reported higher PWB [45].

However, and even more interestingly, the present study confirmed and extended previous findings on the impact of cognitive variables on PWB [17,18], showing that decline in inhibitory abilities, but not in working memory or cognitive flexibility, negatively affects the dimension of personal growth of PWB. Thus, although the efficiency of the executive system emerged to be a fundamental variable in the prediction of PWB during the last decades of the life span [21,22,23,24], here only inhibition was found to play a key role in predicting the participants’ perceived PWB, and more specifically their sense of lifelong growth and development as a person (Personal Growth), even after controlling for age and educational level. 

Moreover, lower interference times, and hence higher ability to inhibit irrelevant stimuli, were associated with higher Personal Growth, and vice versa: older individuals who could efficiently inhibit automatic responses showed higher levels of perceived personal growth. One explanation for such a result may be that the ability to inhibit automatic responses is adaptive in everyday life situations of older people. Indeed, given that older people constantly face challenging problems and situations due to the changes occurring in their lives, beyond their cognitive efficiency, it appears that inhibiting automatic responses (that may no longer be useful or appropriate in this phase of life) can help them to adapt better to changes, accepting new situations, and thus increasing a personal growth. Moreover, considering their lower cognitive efficiency [46] and a general decline in EF abilities [37], older people need to use more controlled rather than automatic mechanisms to adapt to their environment, contributing to the feeling of personal growth. 

Contrary to our hypothesis, no effects of either flexibility or working memory on Personal Growth, as well as on other PWB dimensions, were found. These null effects, on the one hand, might highlight that inhibitory abilities specifically play a key role in favoring adaptation in aging and then on PWB perceived by older adults (also considering that inhibitory abilities are very prone to decline even in healthy aging, e.g., [47,48,49]. On the other hand, it might be hypothesized that these results have been influenced by the limited sample size or by the fact that significant mediators might be neglected in the present research (such as perceived sense of control as evidenced in [24]). Further research is therefore needed to exclude the role of working memory and flexibility for efficient aging. 

This highlights that, even if these results are encouraging, more research is necessary to obtain more robust evidence. A higher number of participants would also be useful to study the extent to which inhibitory abilities might mediate or moderate the relationships between age and well-being.

Further, considering that different studies showed an association between PWB and successful aging [4,11], the understanding of which variables can affect well-being in older adults is critical. To deepen and extend the results reported here, future studies might also examine how different types of inhibitory control are differentially involved in the relationships with PWB, such as reactive (i.e., abrupt stopping of an already planned or initiated action) or the proactive inhibition [50] (i.e., upcoming stop process that can be anticipated as a result of environmental factors). Yet, it should also be clarified whether higher EFs (i.e., reasoning, problem solving, and planning [25]) or other specific cognitive functions can mediate the association between PWB and inhibition.

Additionally, these notions might be pivotal for the construction and planning of effective preventive interventions, which might have a profound positive impact on the mental and physical health of older adults. Several studies have proposed that better executive functioning might play a protective role by increasing adaptive behavior through compensatory processes [51,52,53]. Behavioral and cognitive interventions, that involve executive functioning as a target, were indeed proved to have effects on mental well-being in the older population (e.g., [54,55,56,57,58,59]). In accordance with our results, future interventions might target and promote more efficient inhibitory skills in older people to impact their ability to adapt to the challenges imposed by aging. This might help them, in turn, to experience more purpose and a sense of direction in their lives. Likewise, psychological interventions that target the acceptance of physiological declines due to the aging processes (and of this cognitive capacity specifically) and the promotion of psychological wellbeing might also be useful.

Finally, two other significant points can be highlighted. On the one hand, given that an Italian sample of older adults was enrolled, further studies must be conducted to confirm and generalize the results to samples with different cultural backgrounds, since previous research showed that both culture and socio-economic status have an impact on perceived PWB (e.g., [11,60,61]). On the other hand, these data were collected prior to the spread of COVID-19, which might have had an influence on older adults PWB and on the relationships between the variables considered in this study. However, some results collected during the COVID-19 outbreak confirm the negative relationship found here between age and personal growth [60]. Future studies might consider verifying this issue.

To conclude, it is also important to highlight that longitudinal studies that consider the interaction of multiple variables, such as psychological, biological, cognitive, and environmental (e.g., cultural) ones, would be more effective in predicting perceived PWB. This would help the scientific community to understand whether certain variables might be considered as risk or protective factors and help caregivers, experts, and institutions to promote mental health through targeted prevention and or/stimulation programs.

## 5. Conclusions

EF efficiency, and in particular the ability to inhibit automatic responses, was proven to be pivotal in the context of successful aging because it allows older subjects to adapt to their changing abilities and environments. Specifically, higher inhibitory abilities were found associated with the perception of higher personal growth of PWB. Thus, tailored preventive interventions aimed to enhance inhibition skills appear to have potential significant clinical implications for older adults by improving their PWB, and in turn, their quality of life. Investing in mental health and in psychological well-being will indeed have short- and long-term benefits on the health prognosis of older people, increasing their quality of life and preventing and/or mitigating the potential development of chronic or neurodegenerative diseases.

## Figures and Tables

**Table 1:** Means (M) and Standard Deviations (SD) of the considered variables.

	M	SD
MoCA	24.0	3.06
PWB_Autonomy	61.4	9.47
PWB_Personal Growth	65.8	6.09
PWB_Environmental Mastery	64.7	6.64
PWB_Positive Relation Others	61.2	9.51
PWB_Purpose In Life	63.3	6.78
PWB_Self Acceptance	62.8	9.10
Digit Span Backwards	4.20	1.20
Trail Making Test B-A	115 s	104 s
Stroop_Time interference	32.7 s	24.5 s

**Table 2:** Independent sample t-tests to control for gender differences in perceived PWB and executive functioning.

	Statistics	df	*p*
PWB_Autonomy	−1.371	49	0.177
PWB_Environmental Mastery	−0.202	49	0.841
PWB_Personal Growth	0.594	49	0.555
PWB_Positive Relation Others	−1.943	49	0.058
PWB_Purpose In Life	−0.398	49	0.692
PWB_Self Acceptance	−2.150	49	0.037 *
Digit Span Backwards	0.019	49	0.985
Trail Making Test B-A	−0.426	49	0.672
Stroop_Time interference	−0.785	49	0.436

* *p* < 0.05; all significant results are reported in bold. df = degrees of freedom.

**Table 3:** Two-tailed correlations between psychological well-being (PWB), age and the core executive functions. AUT = Autonomy; EM = Environmental Mastery, PG = Personal Growth; PRO = Positive Relations with Others; PIL = Purpose in Life, and SA = Self-Acceptance; EDU = educational level. + = square root transformed.

		PWB _AUT+	PWB_ EM	PWB_ PG	PWB_ PRO	PWB_ PIL	PWB_ SA	AGE	EDU	DIGIT_ BACK	TMT_ B-A	STROOP_ IT
**PWBAUT+**	r	—										
	*p*-value	—										
**PWB_EM**	r	0.437 **	—									
	*p*-value	**0.001**	—									
**PWB_PG**	r	0.170	0.525 ***	—								
	*p*-value	0.233	**<0.001**	—								
**PWB_PRO**	r	0.270	0.593 ***	0.310 *	—							
	*p*-value	0.055	**<0.001**	**0.027**	—							
**PWB_PIL**	r	0.363 *	0.588 ***	0.485 ***	0.564 ***	—						
	*p*-value	**0.009**	**<0.001**	**<0.001**	**<0.001**	—						
**PWB_SA**	r	0.359 *	0.648 ***	0.389 **	0.615 ***	0.632 ***	—					
	*p*-value	**0.010**	<0.001	**0.005**	<0.001	<0.001	—					
**AGE**	r	0.083	0.099	−0.331 *	0.148	−0.048	0.103	—				
	*p*-value	0.561	0.490	**0.018**	0.299	0.739	0.470	—				
**EDU**	r	0.005	0.096	0.246	−0.067	0.077	−0.040	−0.422 **	—			
	*p*-value	0.975	0.503	0.082	0.641	0.593	0.782	**0.002**	—			
**DIGIT_** **BACK**	r	−0.106	0.088	0.121	0.094	−0.019	0.056	−0.154	0.227	—		
	*p*-value	0.461	0.539	0.396	0.512	0.895	0.696	0.280	0.109	—		
**TMT_B-A**	r	−0.095	−0.053	−0.185	−0.014	−0.099	−0.031	0.463 ***	−0.356 *	−0.406 **	—	
	*p*-value	0.507	0.711	0.195	0.922	0.488	0.830	**<0.001**	**0.010**	**0.003**	—	
**STROOP_IT**	r	−0.051	−0.170	−0.513 ***	0.077	−0.053	−0.064	0.492 ***	−0.335 *	−0.319 *	0.451 ***	—
	*p*-value	0.725	0.233	**<0.001**	0.589	0.713	0.654	**<0.001**	**0.016**	**0.022**	**<0.001**	—

* *p* < 0.05, ** *p* < 0.01, *** *p* < 0.001; all significant results are reported in bold.

**Table 4:** Hierarchical regression with PWB_PG (personal growth) as VD, age and EDU (educational level) as control variables in the first block and the three core executive functions (Digit backwards, TMT B-A and the Stroop’ time interference) as predictors in the second block. *N* = 51; SE = Standard error; Adj. = Adjusted.

		Unstandardized Coefficients		Standardized Coefficients						
Step	Predictors	B	SE	Beta	*p*	R^2^	Adj. R^2^	R^2^ Change	F	*p*
**1**						0.123	0.087	-	3.38	0.042 *
	Age	−0.233	0.125	−0.276	0.070					
	Edu	0.176	0.202	0.130	0.389					
**2**						0.283	0.204	0.160	3.56	0.009 **
	Age	−0.092	0.134	−0.110	0.495					
	Edu	0.108	0.195	0.080	0.581					
	Digit_back	−0.139	0.718	−0.027	0.847					
	TMT_B-A	0.006	0.009	0.103	0.519					
	Stroop_TI	−0.121	0.038	−0.487	0.003 **					

* *p* < 0.05; ** *p* < 0.01; all significant results are reported in bold.

## Data Availability

The dataset will be made available upon requests.
